# Chalcone: A Promising Bioactive Scaffold in Medicinal Chemistry

**DOI:** 10.3390/ph15101250

**Published:** 2022-10-11

**Authors:** Gayathri Rajendran, Deepu Bhanu, Baladhandapani Aruchamy, Prasanna Ramani, Nanjan Pandurangan, Kondapa Naidu Bobba, Eun Jung Oh, Ho Yun Chung, Prakash Gangadaran, Byeong-Cheol Ahn

**Affiliations:** 1 Dhanvanthri Laboratory, Department of Sciences, Amrita School of Physical Sciences, Amrita Vishwa Vidyapeetham, Coimbatore 641112, India; 2 Center of Excellence in Advanced Materials & Green Technologies (CoE–AMGT), Amrita School of Engineering, Amrita Vishwa Vidyapeetham, Coimbatore 641112, India; 3 Department of Sciences, Amrita School of Arts and Sciences, Mysuru Campus, Amrita Vishwa Vidyapeetham, Mysuru 570026, India; 4 Department of Radiology and Biomedical Imaging, University of California (San Francisco), San Francisco, CA 94143, USA; 5 Department of Plastic and Reconstructive Surgery, CMRI, School of Medicine, Kyungpook National University, Kyungpook National University Hospital, Daegu 41944, Korea; 6 BK21 FOUR KNU Convergence Educational Program of Biomedical Sciences for Creative Future Talents, Department of Biomedical Science, School of Medicine, Kyungpook National University, Daegu 41944, Korea; 7 Department of Nuclear Medicine, School of Medicine, Kyungpook National University, Kyungpook National University Hospital, Daegu 41944, Korea

**Keywords:** chalcones, natural sources, Claisen–Schmidt condensation, infectious diseases, non-infectious diseases, structure–activity relationship

## Abstract

Chalcones are a class of privileged scaffolds with high medicinal significance due to the presence of an α,β-unsaturated ketone functionality. Numerous functional modifications of chalcones have been reported, along with their pharmacological behavior. The present review aims to summarize the structures from natural sources, synthesis methods, biological characteristics against infectious and non-infectious diseases, and uses of chalcones over the past decade, and their structure–activity relationship studies are detailed in depth. This critical review provides guidelines for the future design and synthesis of various chalcones. In addition, this could be highly supportive for medicinal chemists to develop more promising candidates for various infectious and non-infectious diseases.

## 1. Introduction

Molecular diversity is a crucial factor for any drug discovery program. The molecules of interest can be obtained through natural or chemical syntheses. Natural products remain a source of unexplored chemotypes (e.g., molecular scaffolds and pharmacophores) and offer many structural fragments for medicinal chemistry. Therefore, natural products are always considered to be invaluable sources of inspiration for drug discovery development. Recently, scientists have been attracted to smaller fragments or scaffolds (~300 Da) due to their unique properties, i.e., effective molecular interactions with the targeted biological receptors for pharmacological properties. These features are very crucial in the area of fragment-based drug design (FBDD). Such exceptional properties are found in flavonoids. Flavonoids are among the largest polyphenols that are widely distributed in fruits, vegetables, and the plant kingdom as secondary metabolites. Flavonoids are classified into chalcones, flavanones, flavones, isoflavones, aurones, neoflavones and biflavones. To date, more than 7500 molecularly diverse flavonoids have been reported. Among flavonoids, chalcones play a prominent role and have been found to be a precursor for the biosynthesis of flavonoids. 

**Figure 1:**
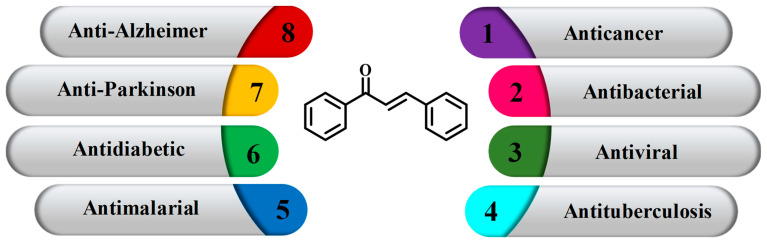
Structure of chalcone and its bioactivities (e.g., anticancer, antibacterial, antiviral, anti-tubercular).

Chalcones have an α,β-unsaturated system with three carbon units as its structural basis [1]. They are known as 1,3-diphenylprop-2-en-1-one, which is implicated in their hydrophobic/hydrophilic nature that has sparked much research—especially in the field of infectious and non-infectious diseases [2]. Chalcones occur in the cis or trans form, of which the trans isomer is more thermodynamically stable than the cis form. Chalcones have been reported to have biological properties such as anticancer [3] antimicrobial [4], antiviral [5], antioxidant [6], anti-Alzheimer’s [7], antitumor [8], antidiabetic [9], anti-Parkinson’s [10], anti-inflammatory [11], and anti-nociceptive [12] effects (Figure 1). After a careful literature search, it has been found that in the past decade many scientific developments took place in chalcone chemistry, which is scattered in nature. Furthermore, heterocyclic rings are known to be bioactive, and incorporating heterocyclic moieties in the structural framework of chalcones will further increase their bioactivity. In recent years, numerous research projects have been completed, and scientists have synthesized various chalcone analogs. Chalcones bearing heterocyclic rings—such as quinoline [13], pyrrole [14], pyridine [15], indole [16], pyrazole [17], benzofuran [18], coumarin [19], isoxazole [20], benzimidazole [21], and azulene [22]—have been reported in the literature. There have also been reports of metallocene-based chalcone analogs such as ruthenocenyl and ferrocenyl chalcones in the literature [23]. Chalcones can also be used for other optical applications, such as fluorescent probes [24]. With this view, the effort was taken to summarize the structure, synthesis, properties, and applications of chalcones over the past decade, and their structure–activity relationship (SAR) studies are described in detail in this review.

## 2. Chalcones from Natural Sources

**Figure 2:**
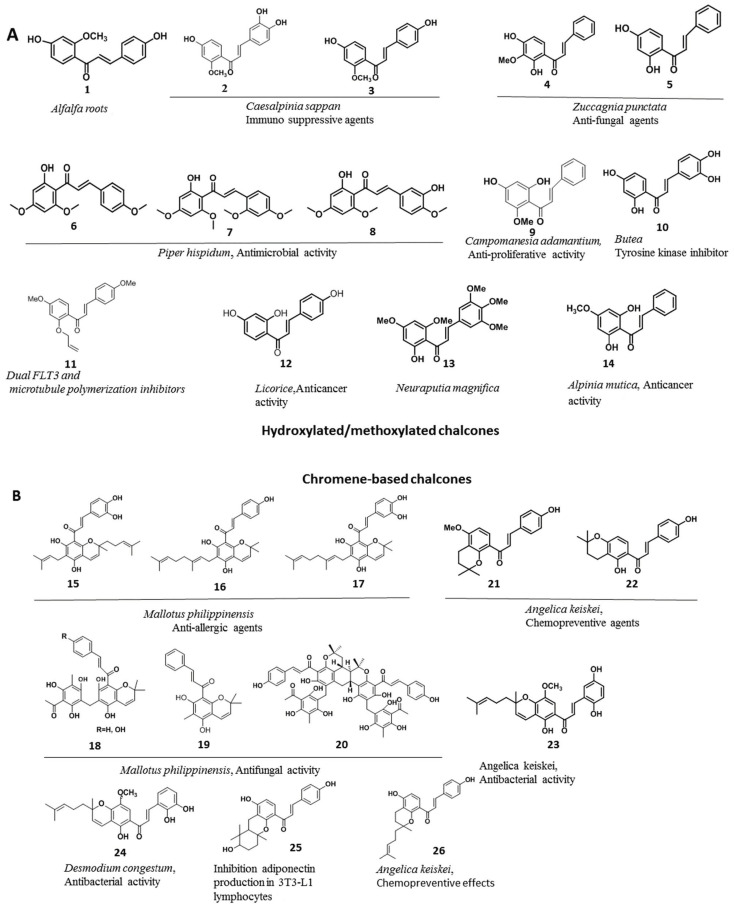

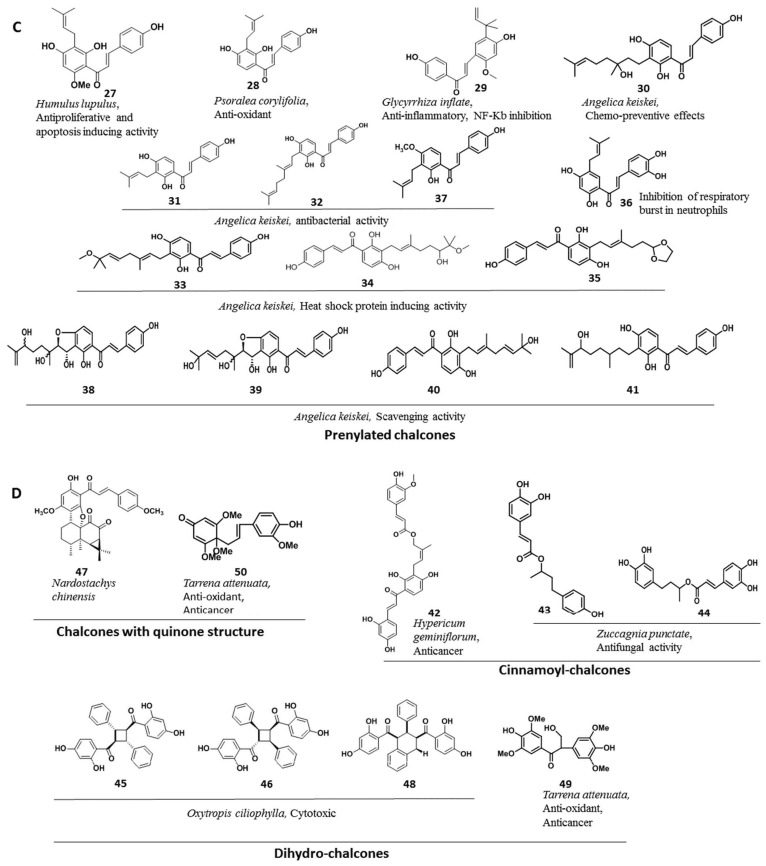
Naturally occurring chalcones: (**A**) hydroxylated and methoxylated chalcones; (**B**) chromene-based chalcones; (**C**) prenylated chalcones; (**D**) quinone-, cinnamoyl-, and dihydro-bearing chalcones.

Chalcones can be biosynthesized by combining phenyl propane and acetate pathways through convergent syntheses. Coumaroyl-CoA is used for making phenylalanine in three enzymatic steps, and malonyl-CoA is synthesized by carboxylation of acetyl-CoA—a central intermediate in the Krebs tricarboxylic acid cycle. The chalcones are further classified based on their structure; for example, the compounds that usually occur in plants are commonly hydroxylated or methoxylated (Figure 2A); glycosylated and prenylated forms are found less frequently (Figure 2C), as are geranylated chromene-based derivatives (Figure 2B) and dihydroquinone-based chalcones (Figure 2D). Herbs that contain chalcones have been used as medicines in the past to treat a variety of illnesses. Chalcones can be found in large quantities in various plants, including *Butea monosperma*, *Desmodium gangeticum*, *Humulus lupulus*, *Helichrysum rugulosum*, *Neoraputia magnifica*, *Angelica keiskei*, *Piper hispidum*, *Tarenna attenuata*, and *Calythropis aurea* [12,25,26,27,28,29,30,31,32,33,34,35,36,37,38,39,40,41,42,43,44,45,46,47,48,49,50,51,52,53,54,55,56,57]. 

## 3. Synthesis of Chalcones

Chalcones possess an α,β-unsaturated ketone with a diphenyl substitution, and their other functionality makes them unique as a scaffold with multifunctional biological properties. Procurement of chalcones from natural sources often entails logistical problems. Thus, they are chemically synthesized through the following methods: 

### 3.1. Conventional Synthesis of Chalcones

Generally, chalcones are synthesized from an aldehyde and acetophenone through Claisen–Schmidt condensation in the presence of an acid or base. Numerous conditions have been established for synthesizing chalcones, as detailed below. 

**Scheme 1:**
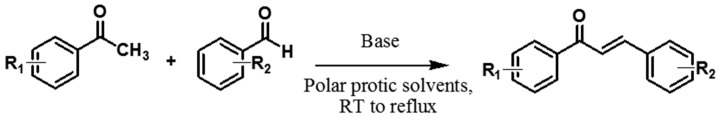
Chalcone synthesis via Claisen–Schmidt condensation.

In the Claisen–Schmidt condensation reaction (Scheme 1), different bases—such as NaOH, KOH, LiOH, Ba(OH)_2_, and organic bases (i.e., piperidine and pyridine) have been used to facilitate the synthesis of chalcones. Likewise, acidic reagents such as HCl and AlCl_3_ have also been established. In both conditions, the yield of chalcone is comparatively less, with an average yield of 50–60% based on the substitutions

Later, to increase the yield, different catalysts were also tried [58,59]. For example, materials such as chitosan [60], Al–Mg hydrotalcite [61], and Cs-pollucite nanozeolite modified with organosilane [62] have been used for the reaction. The acid-based catalyst, i.e., BF_3_-Et_2_O, has many advantages over others, with the yield of the chalcone increasing drastically, which is also helpful for the ease of production [2]. Similarly, other materials—such as activated carbons [63], nanoporous AISBA-15 [64], cesium salt of 12-tungstophoric acid [65], ionic liquids [66], protonated aluminate mesoporous silica nanomaterials [67,68], (MWCNTs)-COOH-CeO_2_ hybrids [69], modified fluorapatite [68], Fe_3_O_4_-MOF core–shell magnetic microspheres [70], nanosized ZnWO_4_ [71], and graphene-supported ZnO nanoparticles [72]—have also been established. BF_3_-etherate can be used as a better catalyst for chalcone synthesis, as the products are obtained with higher yields—in the range of 75–96%. Using heterogeneous catalysts also yields chalcones in higher amounts, where the catalysts can be reused for many cycles of the reaction.

### 3.2. Greener Approaches for the Synthesis of Chalcones

#### 3.2.1. Microwave-Assisted Method

Based on the disadvantages of the conventional methods, microwave-assisted synthesis has been developed for synthesizing chalcones. The main advantage of the microwave-assisted method is that the reaction is usually faster. For example, a few heteroaromatic chalcones can be yielded in 3–5 min without using solvents. Here, the catalysts (the conditions may be either acidic or basic) are combined with the starting materials to form a thick paste, from which the product can be isolated in a soluble form by the elution of catalysts. A series of chalcones have been synthesized using K_2_CO_3_ as a base along with microwave irradiation [73]. Furthermore, chalcones with pyrazoline moieties have been synthesized at higher yields under acidic conditions [74]. 

#### 3.2.2. Ultrasound-Irradiated Synthesis

Similarly, another strategy—the ultrasound irradiation method—can also be established without solvents. The reaction rate is accelerated by ultrasonic irradiation, leading to the formation of the product in a much smaller amount of time and with a higher yield [75]. The completion of the reaction was shown in about 10 s during the synthesis of thiophene-based chalcones with cesium carbonate as a base [76]. Chalcones were also synthesized in the presence of a zeolite-based catalyst in solvent-free conditions, and the yield was greater than 95% [77].

#### 3.2.3. Grinding Technique

This technique provides an alternate way to synthesize chalcones without solvents. The equivalent quantities of aromatic aldehydes, acetophenone, and the reagent are mixed and ground in a porcelain mortar for about 10 min. The desired product can be isolated after adding cold water through filtration [78]. A series of chalcones with different benzaldehydes and 2-acetyl-2-naphthol were synthesized using a grinding technique, and the products were obtained with a yield from 85 to 95% [79].

### 3.3. Coupling Reactions

**Scheme 2:**
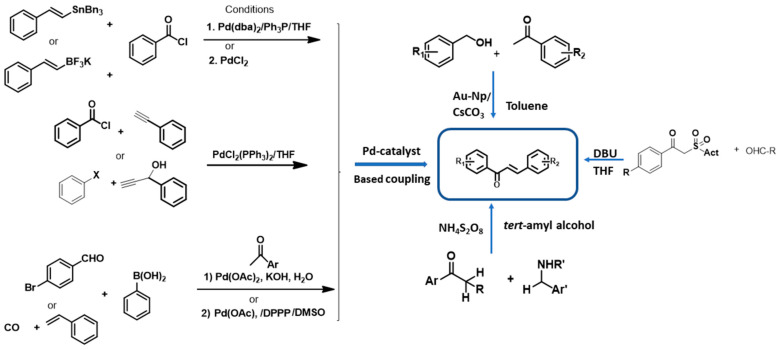
Various methods to synthesize chalcones.

Although chalcones are obtained with a good yield through the greener approaches, they do not generally take the form of multi-substituted chalcones. On the other hand, a coupling reaction could be a good platform for developing chalcone synthesis. Some essential coupling reactions for preparing chalcones are described below (Scheme 2).

Palladium provides an excellent opportunity for developing cross-coupling reactions for synthesizing chalcones; for example, PdCl_2_ is a suitable catalyst for preparing chalcones from potassium styryl trifluoroborates and benzoyl chlorides [80]. Chalcones have been synthesized via silver-catalyzed coupling of cinnamic acids with substituted α-keto acids [81]. In a similar vein, Sonogashira coupling has been utilized to prepare chalcones via the reaction of aryl halides bearing electron-withdrawing groups and prop-2-yl-1-ol [82]. Chalcone derivatives were synthesized using phenylacetylene and benzoyl chloride as starting materials; the reaction followed the same mechanism as Sonogashira coupling [83]. Subsequently, palladium acetate was effectively utilized for the generation of chalcones via the Suzuki reaction under basic conditions. Another method has been developed using palladium acetate through the oxidative carbonylation of boronic acid and styrene via a cross-coupling strategy [84].

**Scheme 3:**
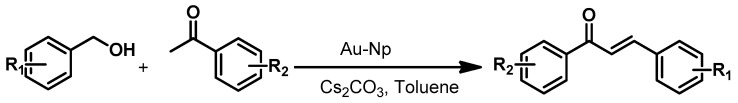
Synthesis of chalcones by aerobic oxidative cross-coupling reaction.

Furthermore, the palladium dibutyl acetate derivative is utilized to develop chalcones through Stille coupling. The reaction involves C-C bond formation between organotin compounds and aryl halides. A new series of chalcones were synthesized by the reaction between organotin compounds and benzoyl chloride [85]. Three different types of coupling reactions have been reported in addition to the palladium coupling reactions. For example, a new series of chalcones were synthesized using gold nanoparticles via a coupling reaction between substituted methyl ketones and aryl alcohols (Scheme 3). The reported reaction is recyclable and gives a good yield of chalcones [86].

**Scheme 4:**
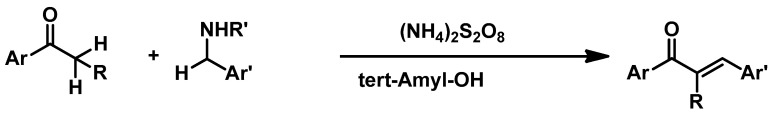
Synthesis of chalcones by cross-dehydrogenative coupling.

Furthermore, a cross-dehydrogenative coupling reaction was developed for chalcones through an enamine-based strategy using a persulfate catalyst. New chalcone analogs were synthesized in 2009 using CDC in the presence of ammonium persulfate as an oxidant instead of metal-based catalysts (Scheme 4) [87]. 

**Scheme 5:**
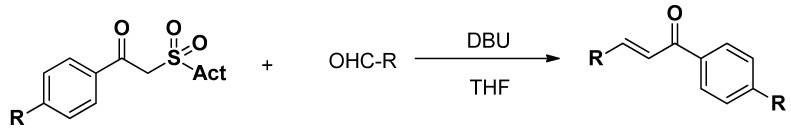
Synthesis of substituted chalcones through Julia–Kocienski olefination.

Further, Julia and Kocienski developed a coupling reaction involving C-C bond formation (Scheme 5). A series of *E*-enones were synthesized using this method, and the product formation was observed with higher yields [88]. The reaction involves β-ketosulfones and aldehyde in the presence of a base. The elimination of sulfur dioxide produces the chalcones. 

### 3.4. Miscellaneous Reactions

**Scheme 6:**
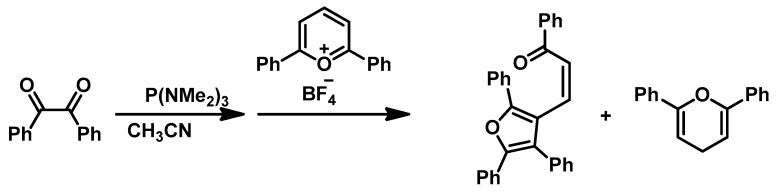
Synthesis of cis-chalcones by reductive (3+2) annulation.

Due to their lower thermodynamic stability, trans-chalcones are synthesized more frequently than cis-chalcones. As a result of this phenomenon, *cis*-chalcones have not been subjected to many applications compared to *trans*-chalcones. *Cis*-chalcones containing furanyl rings were synthesized using reductive (3 + 2) annulation of pyrylium salts with benzil in the presence of P(NMe_2_)_3_ (Scheme 6) [89,90]. 

**Scheme 7:**
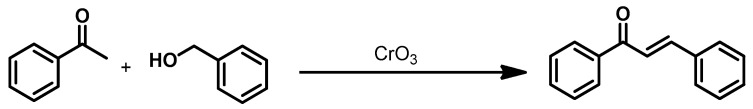
One-pot synthesis of chalcones.

Furthermore, a one-pot reaction has been reported in which the starting materials are directly added to it in the presence of an oxidant. No purification is performed after a single step; thus, one-pot synthesis can be expedited. Phenyl methanol was oxidized into benzaldehyde in the presence of chromium trioxide, which further underwent a condensation reaction with acetophenone to produce chalcones with a good yield (Scheme 7) [90]. 

**Scheme 8:**
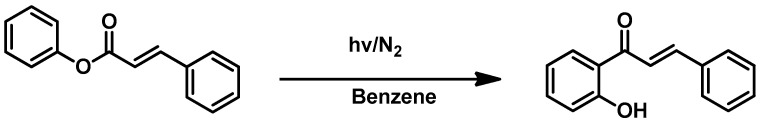
Synthesis of chalcones via photo-Fries rearrangement.

Furthermore, phenyl cinnamate undergoes rearrangement to produce hydroxy-group-bearing chalcones (Scheme 8). The reaction is performed under a nitrogen atmosphere using benzene as the solvent [91].

**Scheme 9:**
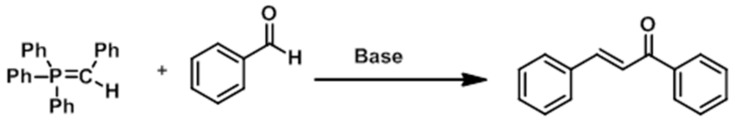
Wittig reaction for the synthesis of chalcones.

Along the same line, the Wittig reaction (Scheme 9) has also been utilized to prepare chalcones. A series of chalcones were synthesized from aromatic aldehydes and arsonium salt in the presence of KF.2H_2_O via the Wittig reaction using the grinding method [92]. The reaction of α-bromoylides with aldehydes in the presence of NBS results in bromo-α,β-unsaturated ketone [93].

## 4. Chalcones against Infectious Diseases

Coronaviruses have affected millions of people across the world. Shigella bacterial infections have also been a significant threat in developing nations, and the mortality rate due to diseases such as TB and malaria is on the rise. Even though many medications are available to treat these diseases, the pathogens often resist them [94]. In recent years, there has been a significant advancement in the study of synthetic and medicinal organic chemistry. Chalcones are considered promising pharmaceuticals, and the following subsections detail their bioactivity against various infectious diseases.

### 4.1. Anti-Tubercular Activity

**Figure 3:**
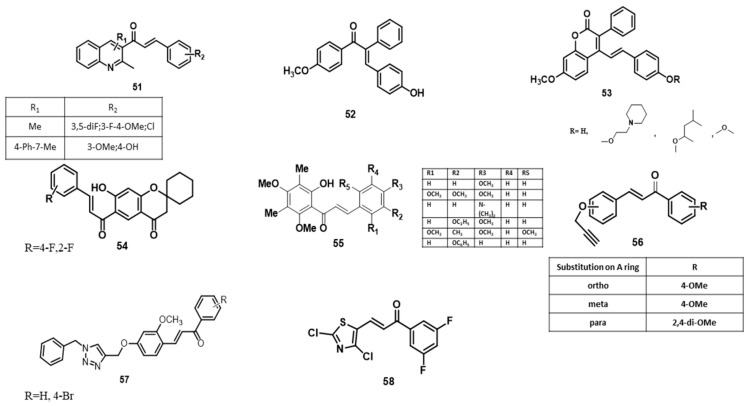
Structures of some chalcones showing anti-tubercular activity.

Tuberculosis (TB) is a contagious illness that primarily affects the lungs. It is ranked as the 7th leading global cause of rising mortality rates. Streptomycin, pyrazinamide, and rifampicin are just a few of the medications created to treat TB [95]. Many chalcone analogs have been tested for anti-tubercular activity, and there have been reports of chalcones with intense anti-tubercular activity [96,97,98] (Figure 3). Quinoline-based chalcone **51** showed anti-tubercular activity against the *M. tuberculosis* H37Rv strain, with better MIC values (10–80 µM) than the standard drug rifampicin [99]. Chalcones with extended phenyl skeletons (**52** and **53**) showed good activity against *Mycobacterium tuberculosis*, with MIC values almost equal to those of standard drugs such as rifampicin and streptomycin. The docking and QSAR study results of these conformationally restricted chalcones also matched the MABA assay results [100,101]. The spirochrome chalcones (**54**) were tested for inhibitory activity against *M. tuberculosis*. Moreover, their docking studies were performed with protein phosphotyrosinase phosphatase B. Docking studies indicated that one of the spirochrome chalcones was bound to the protein at the same pocket region as the natural ligand, and the MIC value indicated that it was more active than the standard drug used in the MABA assay [101].

Seventeen new C-dimethylated chalcones of class **55** were tested for their anti-tubercular activity, and some of them in that class showed higher activity than streptomycin (MIC = 10.75 µM) and ciprofloxacin (MIC = 9.43 µM). The docking results with the *M. tuberculosis* protein tyrosinase phosphatase were analyzed, and compounds with 1,2,3-trimethoxy and 1,2,3,5-tetramethoxy substitutions in the aldehyde ring were more active, which was consistent with results from the MABA assay [102,103]. A class of acetylenic chalcones (**56**) were synthesized and studied for their anti-tubercular activity, with good MIC values (20–100 µM) [103]. Twelve triazole-based chalcones were synthesized, and their activity was studied using the MABA assay and molecular docking (protein 4Y6U). The results indicated that structures (**57**) and scaffolds enhance the activity [96]. Thiazole-based chalcones (**58**) showed more promising activity than the standard drug pyrazinamide, with MIC values of 2.43 µM [104].

### 4.2. Antiviral Activity

**Figure 4:**
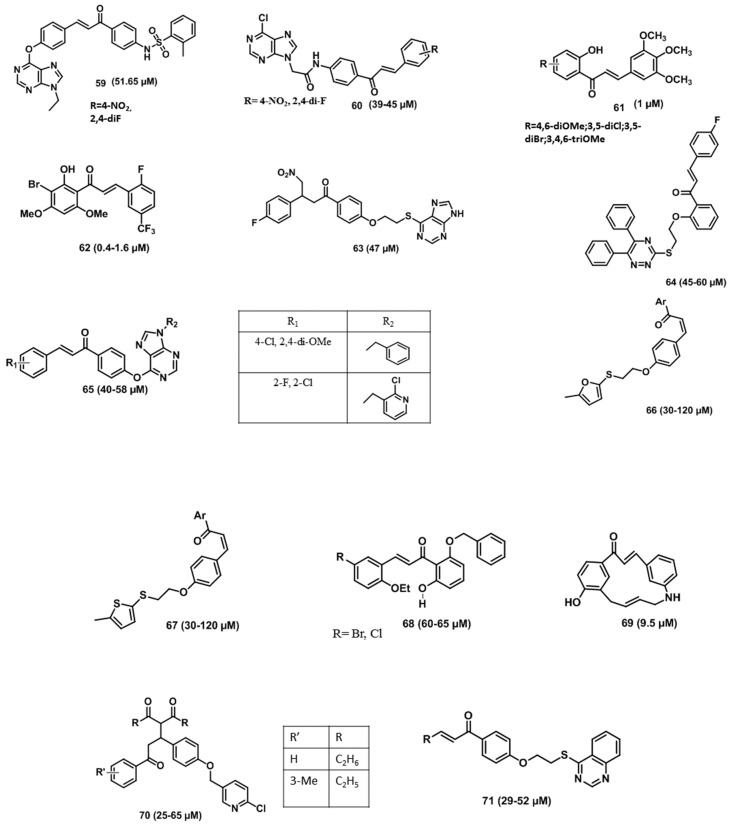
Chalcones with antiviral activity.

Numerous viral infections, including HIV and coronaviruses, cause serious health problems in people all over the world. Numerous plants that naturally contain chalcones have been used to treat viral infections since time immemorial. Some of these are listed in Section 2. The antiviral activity of numerous recently synthesized chalcones has been investigated, and most inhibit the activity of viruses [105,106,107,108]. The structures of some active chalcones are listed in Figure 4. Compound **59** has an EC_50_ value of 51.65 µg/mL, and its docking studies show that it forms four hydrogen bonds in the docking sites of TMV-CP (tobacco mosaic virus coat protein), indicating that it shows good activity against TMV [109]. Chalcone analogs containing purine units were synthesized, and among them, **60**—with a nitro substitution on the para position and difluoro substitution on positions 2 and 4—showed activity against TMV [110]. Chalcone analogs that inhibit the activity of the hepatitis C virus have also been reported (category **61**) [111]. Some natural chalcone derivatives (**62**) were screened for their activity against SARS-CoV-2, with satisfactory EC_50_ values. The results matched the molecular docking studies with the protein RdRp, and the threshold values were calculated [112]. Chalcone derivatives with the purine derivative (**63**) showed a better EC_50_ value than the standard drug ribavirin in inhibiting the tobacco mosaic virus [5]. A 1,2,3-triazine-containing chalcone (**64**) was reported to be active against TMV [113]. Chalcone **65** showed activity against CMV (cucumber mosaic virus) [114]. Chalcones **66** and **67**, containing 1,3,4-oxadiazole and 1,3,4-thiadiazole units, showed efficient activity against TMV and were more effective than ribavirin [115]. Chalcone **68** with an O-benzyl substitution was reported to have anti-HIV activity [108]. Influenza A virus activity was inhibited by compound **69**, which was synthesized via Hoveyda–Grubbs cyclization [116]. Compound **70**—a chalcone derivative bearing malonate and pyrimidine units—showed significant activity against CMV [117]. The 4-thioquinazoline chalcone analog **71** showed potent activity against TMV, with EC_50_ values ranging from 138.1 µM to 154.8 µM, making it much more effective than ribavirin [118].

### 4.3. Antimalarial Activity

**Figure 5:**
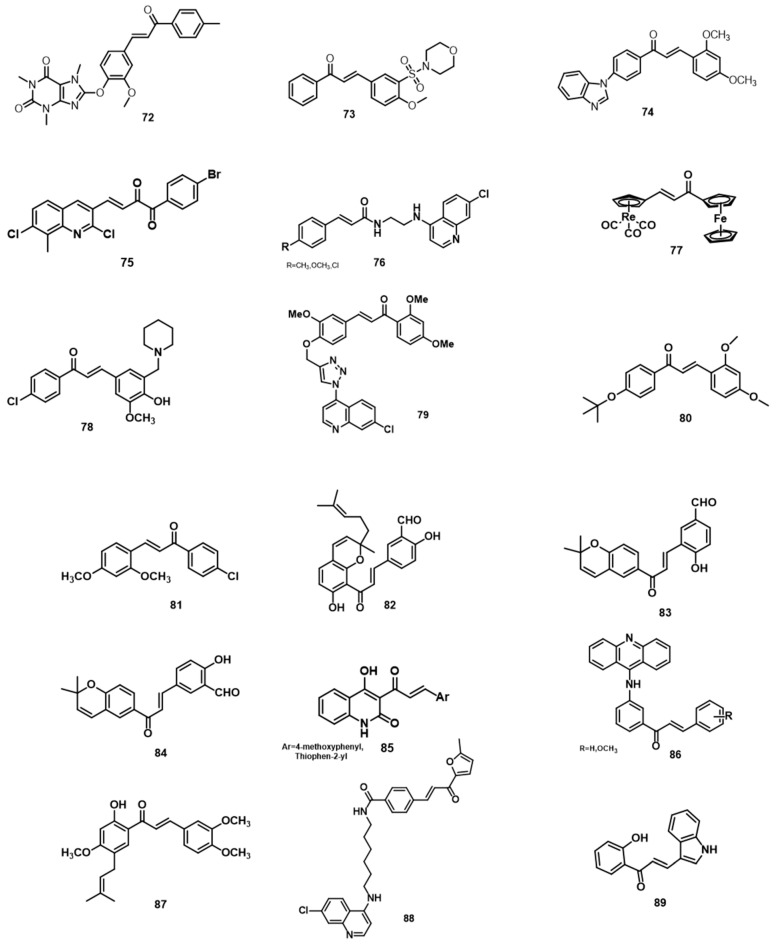
Structures of chalcones with antimalarial activity.

Malaria is caused by a parasite called *Plasmodium falciparum*, which is transmitted by mosquitoes. Antimalarial medications such as chloroquine and artemisinin are readily available. However, modern *P. falciparum* strains are resistant to available medications. Many chalcones that occur naturally have been used to treat malaria [119,120]. Additionally, a large number of synthetically developed chalcones have been tested for their efficacy against various parasite strains. Compound **72** (Figure 5), with a pyrimidine moiety, showed antimalarial activity, with an IC_50_ value of 21.4 µM [121]. A sulfonyl-based 4-methoxychalcone (**73**) could be used as an efficient oral drug against malaria, with an IC_50_ value of 2.06 µM; furthermore, the docking study indicated a comparatively high binding score of −7.3 Kcal/mol [122]. As expected, the imidazole group contributes to the activity, as indicated in compound **74**, which showed potent antimalarial activity, with an IC_50_ value of 1.1 µM [123].

Since the quinolone group has already explored for its antiviral activity, the chalcone with the quinolone scaffold (**75**) showed an IC_50_ value of 0.031 µM and high binding energy with the heme protein [124]. Similarly, another derivative (**76**) showed IC_50_ values in the range of 44.06–59.37 µM and potency against the CQ-3D7 strain [125]. An unusual cyrhetrenyl chalcone (**77**) a derivative containing a ferrocene moiety—showed effective inhibition against strains CQ-3D7 and CQ-W2 [126]. Compound **78**—a chalcone extended with a pyrrolidine unit—was effective against the 3D7 strain. The docking results proved that the molecule shows H-bonding and aromatic interactions with the proteins 1J2I and 1J3K [127]. An azide–alkyne coupling can produce a triazole-bearing chalcone from the combination of quinolone and natural moieties, as in the case of **79**, which was found to be active against three different *P. falciparum* strains: CQ-D10, CQ-Dd2, and CQ-W2 [128]. A normal methoxylated chalcone possesses isopropyl ether linkage, as indicated in compound **80**, and can act as an excellent antimalarial agent with inhibitory activity against two parasitic strains [120,121,129]. Furthermore, chromene-based chalcones **81–84** have inhibitory activity against strains CQ-Dd2, 3D7, and KI [130,131]. Quinolone-based chalcone **85,** synthesized by MW irradiation, was proven to have inhibitory activity against *P. falciparum* [132]. Chalcone **86**, which contains an acridine unit in its structural framework, possesses high efficacy against malaria [133]. Similarly, prenylated chalcone **87** inhibits the activity of the CQ-3D7 strain of *P. falciparum* [134]. A 4-aminoquinolinyl chalcone amide in conjunction with the furano derivative (**88**) was active against two strains, with IC_50_ values ranging from 0.004 µM to 0.07 µM [135]. Furthermore, **89** is an indole-moiety-containing chalcone that is active against malaria, with an IC_50_ value of 2.5 µM [136].

### 4.4. Antibacterial Activity

**Figure 6:**
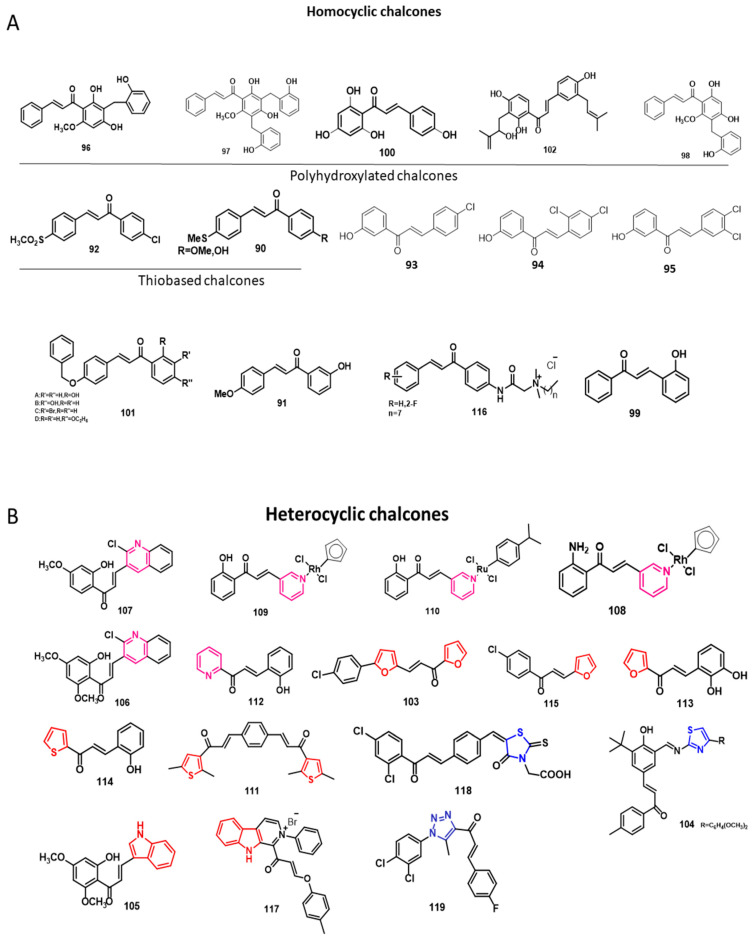
Chalcones with anti-bacterial activity: (**A**) homocyclic chalcones; (**B**) heterocyclic chalcones.

One of the leading health problems affecting human populations is bacterial infection. Pathogens becoming more resistant to antibiotics have caused the effectiveness of current antibiotics to decline. Both natural and artificial chalcones have demonstrated activity against some Gram-positive and Gram-negative strains [137,138]. Biofouling is one of the significant problems in marine-based product development. The chalcones shown in Figure 6 are active against different bacterial strains. In one of the studies, the organism *V. natriegens* (MD6) and other marine strains (*P. fluorescens* and *B. flexus*) were taken for antibacterial studies. The compounds **90**, **91** (0.31–0.61 µM), and **92** (0.0.25–0.195 µM) were reported as having suitable microbial activities against all three organisms and could be used as anti-biofouling agents against marine microorganisms. [139]. Of these compounds, the thio-based chalcone (**90**) was more effective (0.058–0.22 µM) in inhibiting biofouling strains through cell membrane disruption. Chalcones **93**, **94**, and **95** were proven to have good activity, with MIC values in the range of 0.78–1.56 µM [140]. On the other hand, polyhydroxylated chalcones bearing hydroxy groups—**96**–**98**, **100** (62.5–250 µM), and **102** (6.25–25 µM)—were active against methicillin-resistant *Staphylococcus aureus* [141]. Another polyhydroxylated chalcone (**101**) showed enhanced inhibitory activity against Gram-positive organisms (0.1–0.717 µM) such as *Bacillus subtilis* and *Staphylococcus aureus*, as well as the Gram-negative bacteria *Pseudomonas aeruginosa*, *Escherichia coli*, *Salmonella typhi*, and *Proteus vulgaris* [142]. The natural chalcone **102** was reported to be active against two Gram-positive bacteria [143].

The extensive analysis of bacterial studies proves that heterocyclic chalcones are superior to homocyclic chalcones. The *trans*-chalcone containing a difurano ring (**103**) (28 µM) potentially better inhibits the activity of *S. aureus* compared to the standard drug amoxicillin [144]. The thiazole-containing chalcone **104** (1.4 µM) is similar to vancomycin against *S. aureus* [145]. Three nitrogen-heterocycle-bearing chalcones—**105**, **106**, and **107** (20 µM)—act against the Gram-negative bacterium *S. dysenteriae* and two Gram-positive bacteria (*S. Typhi* and *S aureus*) [146]. Compounds **108**, **109**, and **110** are metal complexes formed by chalcones that show inhibitory activity against *E. coli*, *S. aureus*, and *K. pneumonia* (2–52 µM) [147]. Compound **111** is a bis-chalcone-bearing thiophene unit that shows an MIC value (10–12.5 µM) better than that of the standard [148]. Chalcones **112** (128 µM), **113** (128 µM), and **114** (32 µM) are active against *S. aureus* [149]. Compound **115**, with an MIC value of 2 µM, has an inhibitory activity similar to that of norfloxacin [150]. A cationic chalcone (**116**) inhibited the activity of *S. aureus* and MRSA strains, with MIC values ranging from 0.25 to 1 µM [151]. A β-carboline-linked chalcone (**117**) inhibited the activity of *S. aureus*, with an MIC of 440 µM and a ZOI of 15 mm [152,153]. A chalcone bearing a rhodamine-3-acetic acid unit (**118**) showed similar inhibitory activity (2 µM) to norfloxacin against *S. aureus* [153]. Compound **119** can be used as an efficient antibacterial agent with inhibitory activity against four different bacterial strains: *Pseudomonas aeruginosa* (44.06 mM), *Escherichia coli* (55.83 mM), *Staphylococcus aureus* (44.59 mM), and *Bacillus subtilis* (79.76 mM) [154].

## 5. Chalcones for Non-Infectious Diseases

Contact with an infected person does not result in the transmission of non-infectious diseases. They develop as a result of people’s changing lifestyles. These illnesses are challenging to diagnose, and if they are not found at an early stage there are no highly effective medicines available to treat them. Chalcones exhibit activity against cancer, diabetes, and other non-contagious illnesses. In this section, a number of the data from the literature are thoroughly discussed.

### 5.1. Anti-Alzheimer’s Activity

**Figure 7:**
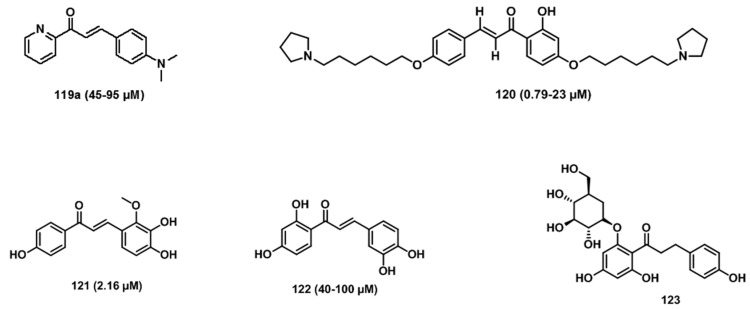
Chalcones for Alzheimer’s disease.

Alzheimer’s disease is a neurological condition that causes memory loss in people—especially those over 65 years old. It also results in other behavioral problems and a decline in language and orientation abilities. The factors vital to its cause are oxidative stress, acetylcholinesterase (AChE), and Aβ1-42 (amyloid beta) aggregation [155,156,157,158,159,160]. Compound **119a** (Figure 7) showed promising activity, inhibiting the aggregation of Aβ1-42 [161,162]. The docking studies on newly synthesized chalcone-*O*-alkylamine analogs were performed with acetylcholinesterase, butyrylcholinesterase (BuChE), and Aβ1-42. It was found that compound **120** was bound to the same pocket region as the natural ligand. Moreover, the in vitro analysis showed that it could cross the BBB and inhibit MAO-B and BACE-1 in the treatment of neurodegenerative disorders [162]. A naturally derived chalcone—L licochalcone B (compound **121**)—exhibits potent anti-Alzheimer’s activity [7]. Butein (compound **122**) [163] and phloridzin (compound **123**) [164] are some chalcones or derivatives occurring in nature, showing anti-Alzheimer’s activity as they induce oxidative stress.

### 5.2. Anticancer Activity

Cancer is one of the most serious illnesses and the leading cause of death worldwide. It is introduced by uncontrolled gene mutation or unregulated cell division in the body. Although drugs with minimal side effects have not yet been created, many chemotherapeutic drugs have been developed in recent years. Chalcones are pharmacologically significant molecules [165,166]. Numerous recently created chalcones exhibit activity against several cancer cell lines, such as MCF-7, A549, and PC3 [166,167,168,169]. 

#### 5.2.1. Anti-Breast-Cancer Activity

**Figure 8:**
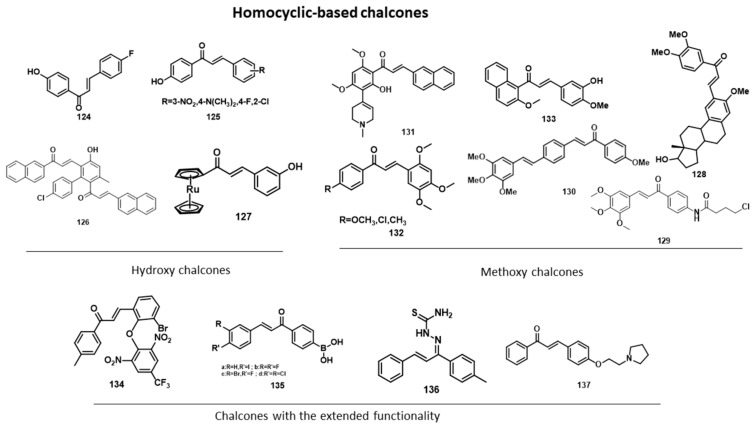
Homocyclic chalcones with anti-breast-cancer activity [170,171,172,173,174,175,176,177,178,179,180,181,182,183].

**Figure 9:**
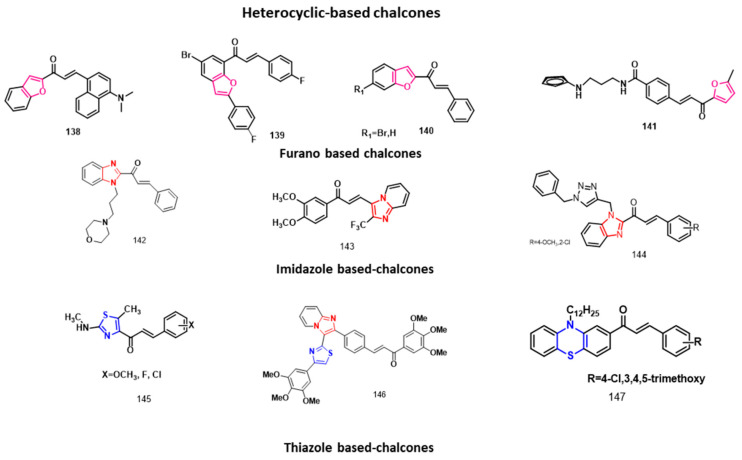

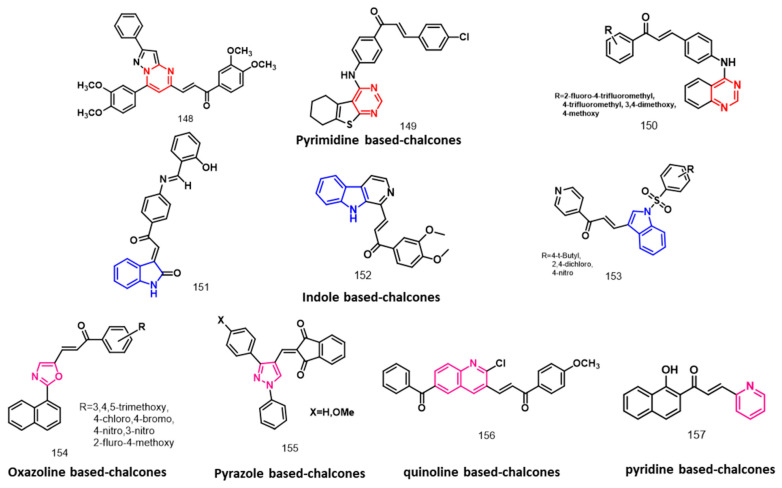
Heterocyclic chalcones with anti-breast-cancer activity [23,184,185,186,187,188,189,190,191,192,193,194,195,196,197,198,199,200,201,202,203,204,205,206,207,208].

Large numbers of chalcones (Figure 8 and Figure 9; Table 1) have been studied for their anti-breast-cancer activities, mainly in MCF-7 cells. Based on their structures, they can be classified into two broad areas: homocyclic and heterocyclic chalcone derivatives. Furthermore, homocyclic chalcones can be divided into three classes: (i) hydroxylated, (ii) methoxylated, and (iii) chalcones with extended functionality. It has been found that chalcones with extended functionality show higher IC_50_ values when compared to other categories, of which the homocyclic chalcone **134** with electron-withdrawing groups such as nitro and trifluoro extensions showed the best IC_50_ value (0.03 µM). 

Similarly, heterocyclic chalcone derivatives are also categorized based on their functional derivatives. For example, a chalcone with a furano derivative (**139**) (IC_50_ = 0.00035 µM) showed excellent inhibitory activity in the nanomolar range compared with other counterparts (i.e., **138, 140,** and **141**). Furthermore, one representation from each of the categories—such as imidazole **143**, IC_50_ = 0.56 µM), thiazole (**146**, IC_50_ = 0.18 µM)), pyrimidine (**150**, IC_50_ = 0.14 µM), and oxazoline (**154**, IC_50_ = 0.35 µM) derivatives—showed inhibitory activity in the range of nanomolar concentrations. However, the indole/quinolone/pyridine classes showed comparatively less activity compared to derivatives **139, 143, 146, 150**, and **154**. Of the listed compounds, heterocyclic derivatives showed comparatively higher anti-breast-cancer activity than homocyclic derivatives. The observation identified that furano derivative **139** with electron-withdrawing ability was the best inhibitor of breast cancer cell lines.

#### 5.2.2. Anti-Lung-Cancer Activity

**Figure 10:**
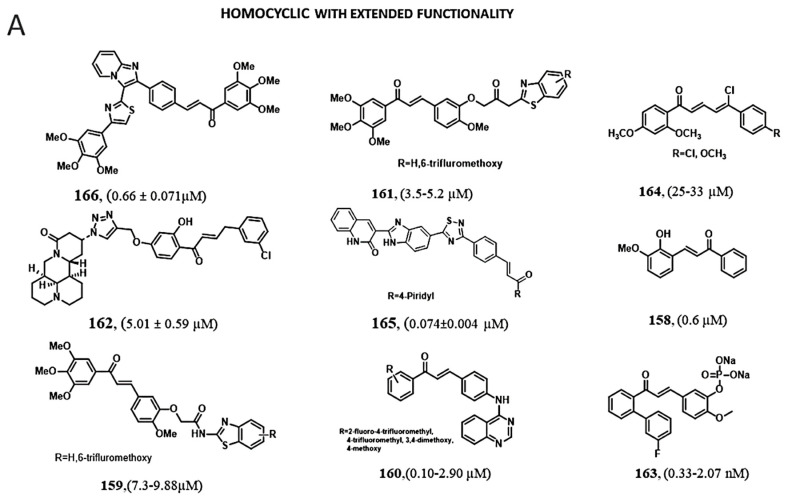

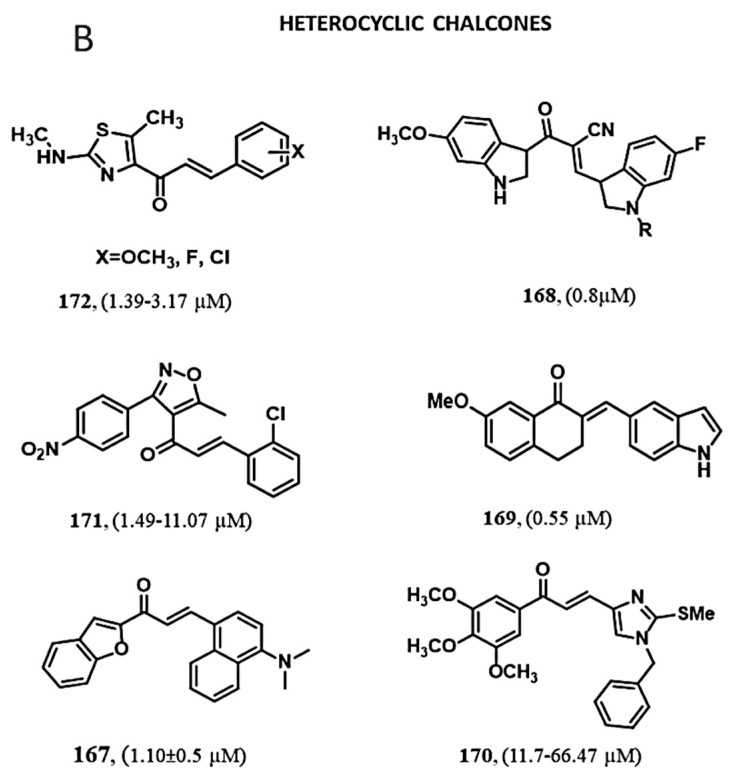
(**A**,**B**) Chalcones with anti-lung-cancer activity and their IC_50_ values [187,193,200,209,210,211,212,213,214,215,216,217].

Furthermore, chalcones have been extended for their anti-lung-cancer activities. In the area, chalcones have been categorized in two ways: (i) chalcones with extended functionality, and (ii) heterocyclic chalcones. In the homocyclic category, compounds **160, 163, 165**, and **166** show inhibitory activities in the nanomolar range. In the heterocyclic category, unlike other inhibitory activities, only a few compounds (**168** and **169**) showed the best inhibitory activities in the nanomolar range. Amongst the compounds reported, the chalcones with extended heterocyclic functionality showed the best inhibitory activity, with IC_50_ values of 0.33–2.07 nM (Figure 10) in the A549 and HLF cell lines. 

#### 5.2.3. Chalcones with Broad-Spectrum Anticancer Activities

Some of the chalcones in Table 2 indicate a broad spectrum of anticancer activities on different cancer cell lines. The homocyclic chalcones—such as **174, 179, 182, 185**, and **187**—have broad-spectrum anticancer activities with moderate IC_50_ values. Furthermore, heterocyclic chalcones (**173, 175, 176, 181**, and **184**) showed lower to larger IC_50_ values, and chalcones with extended hetero functionality (**177, 178, 179, 182, 183,** and **186**) were found to be the best in the broad-spectrum cancer cell lines, as indicated in the table. Moreover, chalcones with nitrogen- and sulfur-containing heterocycles and with methoxy substitutions have been reported to be active against microbes, leukemia, prostate cancer, and colon cancer [218,219,220,221,222,223,224,225,226,227,228,229,230,231,232,233,234,235].

### 5.3. Antidiabetic Activity

**Figure 11:**
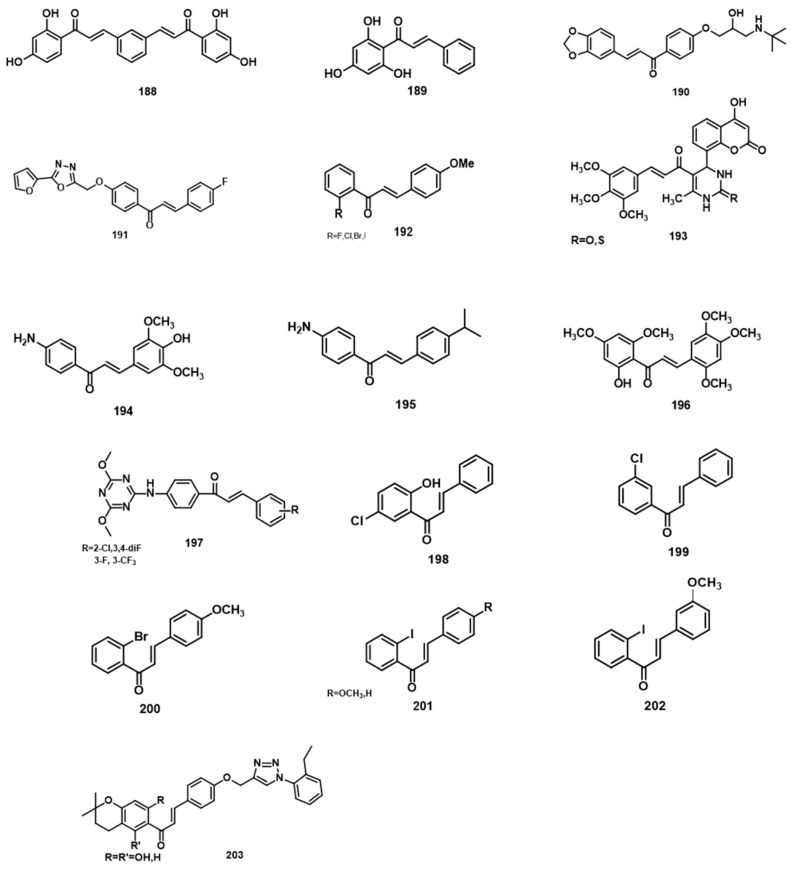
Structures of chalcones with antidiabetic activity.

Diabetes is a metabolic disorder characterized by high blood glucose levels. The inhibition of crucial enzymes—such as α-glucosidase, α-amylase, tyrosinase phosphatase, AMP-kinase, and aldose reductase—can cure hyperglycemia [9,239,240,241]. Compounds such as bischalcone **188** inhibit the activity of α-glucosidase, with an IC_50_ value of 23.7 µM [242]. Compound **189** (0.92 µM) is a trihydroxy chalcone that is effective against diabetes as it inhibits AMP-kinase activity [243]. A chalcone with an aryloxy propylamine unit (**190**) has been reported as an effective antidiabetic agent, inhibiting type 2 diabetes in rats [244]. Compound **191** shows good inhibitory activity against PTP1B [245]. Compound **192**, with the presence of methoxy and halogen groups, exhibits activity against diabetes [238]. Chalcone **193** (0.5–2.5 µM) was active against type 2 diabetes even though its docking results were not high as expected [239]. Compounds **194** and **195** are aminochalcones, while **196** is a naturally derived hydroxychalcone showing inhibitory activity against the enzymes peptidase, α-glucosidase, and PPAR, which decrease the blood glucose levels [9]. A triazine-bearing chalcone (**197**) showed potent antidiabetic activity [246]. Compounds **198–202** (Figure 11) exhibit higher antidiabetic activity than the standards rosiglitazone and pioglitazone (250–300 mg/dL) [247]. Chalcone **203** shows inhibitory activity against α-glucosidase, with an IC_50_ value of 67.77 µM [248].

### 5.4. Anti-Parkinson’s Activity

**Figure 12:**
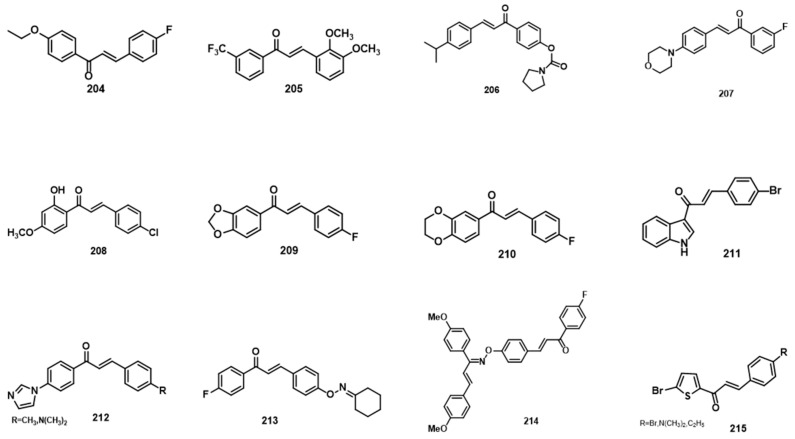
Chalcones showing anti-Parkinson’s activity.

Parkinson’s disease is a neurological disorder involving the loss of dopamine neurons in the nigrostriatal pathway and decreased levels of neurotransmitters. Coordination, mental health, and mobility are all impacted by this. Chalcones have been found to inhibit MAO-B receptors in the literature (Figure 12) [10,249]. Compound **204** is an ethoxylated chalcone that can bind reversibly with the MAO-B pocket site, with an IC_50_ value of 0.53 µM [10]. The trifluoromethyl-substituted chalcone **205** is a suitable hMAO-B inhibitor [250]. Compounds **206** and **207** are active against Parkinson’s disease, with IC_50_ values of 0.29 µM and 0.087 µM [251]. Chalcone **208** can act as a good Parkinson’s drug, with an IC_50_ value of 0.0044 µM [252]. Compounds **209** and **210** (1–108 µM) are potent MAO-B inhibitors [253]. Compound **211** (1.20 µM) is an indole-based chalcone that can easily cross the BBB, and the results have been analyzed via PAMPA assay [254]. An imidazole-containing chalcone, **212** (20–100 µM), acts as both an MAO-A and MAO-B inhibitor [255]. Chalcones bearing oxime ethers—**213** (76.8 µM) and **214** (81.8 µM)—and the thienyl chalcone **215** are active against diabetes, and their docking studies indicate that they bind efficiently at the pocket regions of hMAO-B [256].

## 6. SAR Studies

The extensive search and analysis showed that chalcones possess outstanding biological properties with respect to infectious and non-infectious diseases. The first part of the study indicated that 60 new chalcones have been isolated from different families—mainly from Fabaceae, Euphorbiaceae, Cyperaceae, Piperaceae, Lecythidaceae, Annonaceae, Myrtaceae, Umbelliferae, Lauraceae, Rutaceae, Rubiaceae, Caprifoliaceae, and Zingiberaceae. The structures of the reported chalcones in the plants are hydroxylated, methoxylated, a combination of hydroxylated and methoxylated, prenylated, non-prenylated, glycosylated, cinnamoylated, dihydroformylated, and so on. Scarcity and chemotaxonomy lead to the synthesis of chalcones via chemical methods. With this in mind, many synthesis methods have been discovered and listed. However, extensive protection and de-protection strategies are necessary to prepare functional-based chalcones such as hydroxyl-, amino-, and thio-based compounds. Both synthetic (compounds **124** and **125**) and natural chalcones (compounds **1–14**) bearing hydroxyl functional groups demonstrate higher activity in breast cancer cell lines [257,258]. Similarly, halogenated synthetic chalcones and methoxylated chalcones derived from both natural and synthetic sources have proven to be highly active against liver, prostrate, and lung cancer cell lines [259,260,261,262]. In addition, trihydroxylated and methoxylated chalcones showed higher activity than di- and mono-substituted ones [263,264,265].

**Figure 13:**
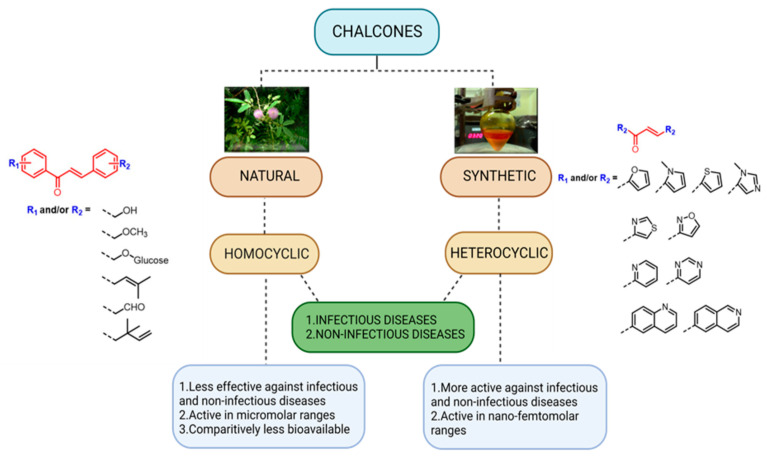
Flowchart depicting the structure–activity relationships of natural and synthetic chalcones.

From this report, it is very much clear that natural analogs of homocyclic chalcones with extended hetero functionality and heterocyclic chalcones possess excellent inhibitory activity against infectious and non-infectious diseases (Figure 13). In a similar vein, naturally occurring chalcones are poorly bioavailable molecules due to their decreased absorption in the intestine. However, the new chalcone categories are yet to be analyzed in terms of this particular property of interest. Similarly, the reported chalcones can be taken to the next level of studies, including animal studies, clinical trials, and much more.

## 7. Conclusions and Perspectives

Chalcones are well-known chemical compounds with high pharmacological importance. They can be of natural or synthetic origin. Many natural chalcones have been used as medicines since ancient times. Since the 1980s, research has been progressing in this particular area. Various routes have been developed for the synthesis of chalcones. The principal method used to synthesize chalcones is Claisen–Schmidt condensation. Other synthetic methodologies include coupling reactions in the presence of organometallic catalysts and rearrangement reactions. Light-enhanced synthesis methodologies have also been reported in the literature. Many studies have been conducted by varying the structural framework of chalcones by incorporating different heterocyclic moieties, and most showed different bioactivities. Metal-incorporated chalcones and their complexes have also been reported. Most chalcones are fluorescent and can be employed as fluorescent probes. Compared to synthetic chalcones, the extraction of natural chalcones often takes a long time and produces low yields. Chalcones obtained from natural sources are homocyclic, demonstrating lower bioactivity when compared to compounds synthesized with heterocyclic units. Many organic reactions have been reported in the literature to obtain a wide variety of substituted chalcones that are employed as anticancer, antidiabetic, antioxidant, antibacterial, anthelmintic, antiulcer, antiviral, insecticidal, and antiprotozoal agents. Therefore, the ability to structurally modify chalcones through synthesis has better advantages than natural chalcones, such as higher yields, easy handling, cost-effectiveness, and much more. The literature indicates that chalcones are most active against cancerous disorders—mostly against breast cancer cell lines, and also against lung cancer. Furthermore, SAR studies indicate that they have good ADMET properties, making them efficient as drug molecules. From this point of view, it is clear that these molecules’ research interest and importance will further increase in the coming years.

## Figures and Tables

**Table 1:** Chalcones with anti-breast-cancer activities.

**Homocyclic chalcone derivatives (breast cancer cell lines; IC_50_ values in µM)**					
Hydroxylated chalcones		Methoxylated chalcones		Chalcones with extended functionality	
**124**	**1.37**	**128**	7.3	**134**	**0.03**
**125**	-	**129**	2.54	**135**	3.5
**126**	4.4	**130**	30.55	**136**	-
**127**	91.4	**131**	23.45	**137**	<10
-	-	**132**	2.2	-	-
-	-	**133**	1.2	-	-
**Heterocyclic chalcone derivatives (breast cancer cell lines; IC_50_ values in µM)**					
Furano-based chalcones		Imidazole-based chalcones		Thiazole-based chalcones	
**138**	2.2	**142**	8.91	**145**	1.97
**139**	**0.00035–0.59**	**143**	**0.56**	**146**	**0.18**
**140**	1.45	**144**	5.89	**147**	12
**141**	1.8	-	-	-	-
Pyrimidine-based chalcones		Indole-based chalcones		Oxazoline/pyrazole/quinoline/pyridine-based chalcones	
**148**	7.4	**151**	31.66	**154**	**0.35**
**149**	6.52	**152**	2.25	**155**	3.9
**150**	**0.14**	**153**	12	**156**	2.32
-	-	-	-	**157**	1.8

**Table 2:** Chalcones with diverse anticancer activities.

Compound Number	Structure of Chalcones	Types of Cancer
**173**	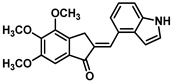	Cervical cancer (0.027 ± 0.01 µM), prostate cancer (0.031 ± 0.05 µM), leukemia (0.031 ± 0.12 µM), lung cancer (0.026 ± 0.03 µM) [236]
**174**	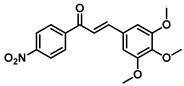	Lung cancer, colon cancer, renal adenocarcinoma, pancreatic carcinoma [237]
**175**	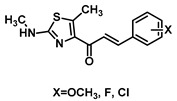	Lung cancer (1.39–3.17 µM), breast cancer (1.97–4.14 µM), hepatocarcinoma (1.56–3.79 µM) [186]
**176**	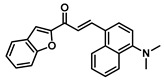	Breast cancer (2.2 ± 0.3 µM), prostate cancer(0.9 ± 0.5 µM), lung cancer (1.10 ± 0.5 µM), pancreatic carcinoma (1.2 ± 0.2 µM) [187]
**177**	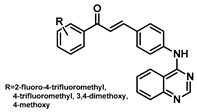	Lung cancer (0.10–2.90 µM), breast cancer (0.14–0.17 µM), colon adenocarcinomas (0.13–2.89 µM) [189]
**178**	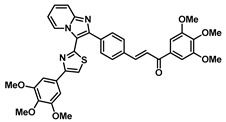	Lung cancer (0.66 ± 0.071 µM), breast cancer (0.18 ± 0.094 µM), prostate carcinoma (1.03 ± 0.45 µM) [193]
**179**	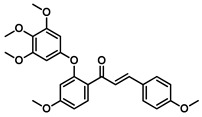	Breast cancer (3.44 ± 0.19 µM), liver carcinoma (4.64 ± 0.23 µM), adenocarcinoma (6.31 ± 0.27 μM) [171]
**180**	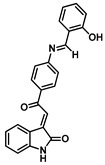	Colon cancer (11.78 µM), breast cancer (31.66 µM), liver cancer (13.95 µM) [194]
**181**	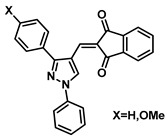	Breast cancer (3.9–4.1 µM), liver cancer (3.8–5.0 µM), colorectal cancer (3.3 µM) [199]
**182**	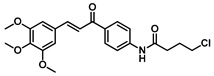	Breast cancer (2.54 µM), colorectal cancer (1.83 µM), gastric carcinoma (1.52 µM) [178]
**183**	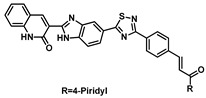	Breast cancer (0.012 ± 0.007 µM), lung cancer (0.074 ± 0.004 µM), colon cancer (0.074 ± 0.004 μM), ovarian cancer (0.083 ± 0.002 μM) [200]
**184**	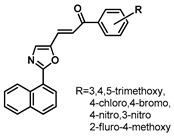	Breast cancer (0.33–0.89 µM), melanoma (0.11–1.28 µM), lung cancer (0.34–7.56 µM) [238]
**185**	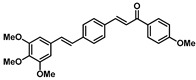	Ovarian carcinoma (6.66 µM), breast cancer (30.55 µM), lung cancer (36.35 µM) [181]
**186**	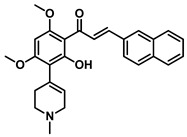	Colon carcinoma (33.31 µM), cervical carcinoma (21.80 µM), breast cancer (23.45 µM), lung cancer (4.28 µM) [206]
**187**	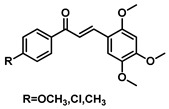	Breast cancer, synovial carcinoma, cervical carcinoma (2.2–4.5 µM) [183]

## Data Availability

Not applicable.
